# The Second Skin: Ecological Role of Epibiotic Biofilms on Marine Organisms

**DOI:** 10.3389/fmicb.2012.00292

**Published:** 2012-08-23

**Authors:** Martin Wahl, Franz Goecke, Antje Labes, Sergey Dobretsov, Florian Weinberger

**Affiliations:** ^1^Department Benthic Ecology, Helmholtz Centre for Ocean Research KielKiel, Germany; ^2^Kieler Wirkstoff-Zentrum at Helmholtz Centre for Ocean Research KielKiel, Germany; ^3^Department Marine Science and Fisheries, Sultan Qaboos UniversityMuscat, Oman

**Keywords:** stress, microbe-macroorganism interaction, modulation of interactions, epibiosis, chemical ecology, biofilm

## Abstract

In the aquatic environment, biofilms on solid surfaces are omnipresent. The outer body surface of marine organisms often represents a highly active interface between host and biofilm. Since biofilms on living surfaces have the capacity to affect the fluxes of information, energy, and matter across the host’s body surface, they have an important ecological potential to modulate the abiotic and biotic interactions of the host. Here we review existing evidence how marine epibiotic biofilms affect their hosts’ ecology by altering the properties of and processes across its outer surfaces. Biofilms have a huge potential to reduce its host’s access to light, gases, and/or nutrients and modulate the host’s interaction with further foulers, consumers, or pathogens. These effects of epibiotic biofilms may intensely interact with environmental conditions. The quality of a biofilm’s impact on the host may vary from detrimental to beneficial according to the identity of the epibiotic partners, the type of interaction considered, and prevailing environmental conditions. The review concludes with some unresolved but important questions and future perspectives.

## Microbial Communities Associated with Macroorganisms in the Sea: Antagonism, Neutralism, Synergism in Epibiosis

In contrast to air, the ocean represents a benign environment for most living organisms: With the exception of some harsh marine environments, the means of physico-chemical properties are generally not far off the optimum of most species and their fluctuations are moderate, rarely exceeding biological tolerance limits. As a consequence, insulating coatings of the epidermis such as hair, feathers, wax are not required in the marine realm. When protective armor against predation or mechanical stress (cuticles, shells, spines, tunics, etc.) is not realized because a species rather relies on escape, hiding, poor palatability or chemical defenses, its outer body surface represents its major physiological interface with the environment. This interface is often delicate serving a multitude of exchange processes with the environment: respiration, exudation of wastes and secondary metabolites, absorption of energetic irradiation or informational signals, uptake of nutrients, and gases, etc. The body surface of a nudibranch, for instance, may be considered the combined equivalent of human skin, eyes, (internalized) lungs, intestine, and kidneys. From an ecological perspective, most interactions among conspecifics, or host/parasite and predator/pray pairs are linked to and controlled by properties of the organism’s body surface. Finally, most environmental stressors such as, e.g., desalination, hypoxia, UV radiation, and pollution are experienced at this functional interface foremost.

The functioning of such delicate interfaces is threatened by fouling, i.e. the settlement of other organisms onto this surface. Such non-trophic association between a basibiont (host) and an epibiont (on-growing organism) is called epibiosis (e.g., Wahl, 1989). The dispersion stages of potential settlers, ranging from bacteria to the propagules of invertebrates or macroalgae are always present in the sea albeit varying in composition and concentration regionally, with depth, and with season. The concentration of the various forms can reach densities per ml of seawater of 10^6^ for bacteria, 10^3^ for microalgae, and 10^2^ for propagules of animals and macroalgae (for references, see Harder, 2009). It is not surprising therefore that any undefended surface is overgrown by micro- and macro-foulers within days or weeks. Such an uncontrolled biotic coverage of an organism’s body surface will have a multitude of, mostly detrimental, consequences for the basibiont: Increased weight and friction, impeded trans-epidermal exchanges, altered color, smell, and contour with multiple consequences. These proximate changes to the host due to epibiosis may lead to a loss of buoyancy, an impediment of motility, a hindrance to mating, or a substantial shift of interactions among species (e.g., Prescott, 1990; Dougherty and Russell, 2005; Wahl, 2008b) and is thought to be the selecting force behind the evolution of a variety of antifouling strategies. While the direct and indirect effects of macro-epibiosis, i.e., the colonization of a basibiont by macroscopic epibionts have been thoroughly studied, the consequences of epibiotic microbial fouling have received substantially less attention. The reasons for this asymmetry of investigative effort are obvious: The presence of epibiotic biofilms (microbes enclosed within an exopolymeric matrix) is less conspicuous, its constituents are not comprehensively described (for the most part, the constituents are inaccessible by common culture techniques and even the recent advent of molecular tools and fast sequencing techniques does not lead to identification of all organisms in the biofilm), and its abundance and compositions seem to be highly variable and dynamic. Furthermore, it is difficult to study the functioning of epibiotic biofilms without the confounding input of the host. Finally, due to their thinness and often negligible biomass the physiological and ecological impact of epibiotic biofilms until recently may have been severely underestimated.

**Figure 1:**
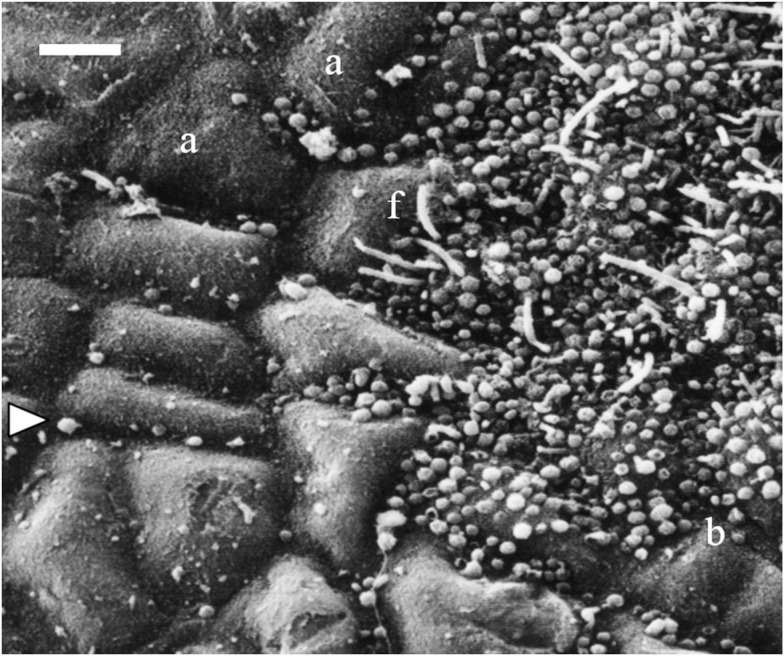
**Scanning electron micrograph showing a partially fouled surface of *Fucus vesiculosus* with unobstructed and masked areas of host tissue**. The left side of the picture shows an apparently clean surface, the algal cells are visible (a) and also few coccoid bacteria (arrow) between them. In contrast, the right side of the picture shows a microbial film with coccoid bacteria (b) and filaments (f) covering the algal cuticle. The photo also illustrates the patchiness of microfouling on one host individual. Scale bar = 5 μm.

Biofilms develop easily at any solid/liquid interface in humid or aqueous environments. By a dynamic equilibrium between settlement of planktonic (“free”) bacteria and detachment of biofilm bacteria the two major bacterial compartments remain connected (Grossart, 2010). Free bacteria are attracted to point sources of organic matter, such as aggregates or organism surfaces rapidly react to appropriate stimuli by attachment and physiological shifts (Grossart and Tang, 2010). These authors describe that biofilm bacteria in comparison to their planktonic life form, are more densely packed by 1–2 orders of magnitude (Figure 1), communicate more intensively, show higher enzymatic activity, growth and production, and exercise more intense lateral gene transfer. At the same time they seem to be more susceptible to predation and infection in the attached life stage. Bacterioplankton is well studied in most regards while knowledge about the biology and ecology of biofilm bacteria is just emerging. Early studies on the role of biofilms stem mostly from the medical and the technical fields. Composition and functioning of biofilms have been thoroughly investigated on internal (and external) surfaces of the human body (plaque, intestinal flora, bacterial fouling on implants: reviewed by Robinson et al., 2010) and on technical surfaces as diverse as sensor heads, reverse osmosis membranes of desalination plants, drink water pipes, or ship hull paints (reviewed in Dürr and Thomason, 2010 and references therein; Railkin, 2004; Flemming, 2009). Apart from clogging, shading, corrosive, and degrading effects, the major interest of many researchers was the fouling-mediating role of biofilms (reviews by Dobretsov et al., 2009; Hadfield, 2011). The consensus of most investigations is that the presence of biofilms alters the substratum physically (wettability, microtopography, consistency) and chemically (alteration of the substratum, degradation of substances released by the substratum, exudation of bacterial metabolic products), and that they have the capacity to modulate (reduce, enhance, select) the recruitment of other bacteria, diatoms, fungi, larvae, or spores. The capacity to hinder further fouling seems to be more prevalent in epibiotic biofilms than in the bacterial assemblage of the water column (Burgess et al., 1999). Whether this is also true for the enhancement of settlement (Hadfield, 2011) is not known. However, the establishment of epibiosis is not a simple process, and various physical and chemical properties of the host surface, as well as interactions among the settlers are determinants of the formation of specific communities (e.g., Wahl et al., 2010; Steinberg et al., 2011).

**Figure 2:**
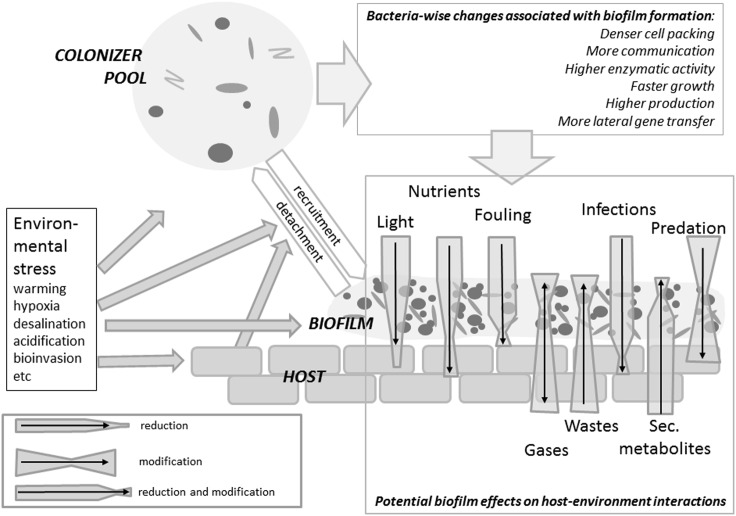
**Summary of biofilm impact on the host varying from detrimental to beneficial effects according to the epibiont’s identity, the type of interaction considered and the environmental conditions**. Via a recruitment/detachment equilibrium – controlled by environmental and host traits – epibiotic bacterial communities are connected to the free water phase. When forming a biofilm, bacteria experience a boost in activity and interactions. The host will experience a certain reduction in irradiation. Fouling, infections and predation will be affected by the presence of a biofilm, but extent and even sign of these effects are context-specific. An algal host will experience a reduction or an enhancement in nutrient availability depending on whether the autotrophic, respectively heterotrophic components prevail in the biofilm. Wastes and secondary metabolites (including infochemicals) may be metabilized by the biofilm.

It is obvious that the multiple possible functions and activities of biofilms (described in later sections and depicted in Figure 2) render their presence on living surfaces everything but trivial. There are probably no marine organisms whose surface is free of epibiotic bacteria and only very few continuously exhibit an almost sterile surface such as some colonial didemnid ascidians (Wahl and Lafargue, 1990). The vast majority of marine organisms bear epibiotic biofilms of variable density and composition (e.g., Lachnit et al., 2009; Grossart, 2010). Considering the diversity of the already known effects, it can be expected that the nature of this biofilm will affect the basibiont’s physiology and ecology in beneficial, detrimental, or ambiguous ways. In fact, since biofilms in form and function are considered almost analogous to multicellular organisms (Steinberg et al., 2011) epibiotic microfouling leads to the replacement of the host’s epidermis as the sole functional interface between host and environment by a new, and functionally different, “tissue,” the epibiotic biofilm. Cells in this biofilm “tissue” interact with each other, exchange metabolites and information, multiply and even produce propagules (“dispersers”) when internal or external conditions degrade (reviewed in Steinberg et al., 2011). The analogy to multicellular organisms, however, is limited by the facts that cells in multispecies biofilms do not share the same genome and that each establishment of a biofilm produces a differently composed “organism” albeit with often similar functionality (Burke et al., 2011a). The following review will give evidence of our still embryonic knowledge on the ecological role of biofilms epibiotic on marine organisms. In this review, we focus on effects the host experiences from this association with a biofilm while being well aware that the interaction is reciprocal and biofilm bacteria are affected by host traits in many regards.

## Bacterial Communities at the Surface of Macroorganisms

In nature, every single macroorganism is found to maintain more or less stable relationships with prokaryotes (McFall-Ngai, 2000, Table 1 for algal hosts). Some core roles of bacteria for the development and evolution of the host have recently been reviewed (Fraune and Bosch, 2010). Most bacteria, and particularly those associated with the surface of other organisms, occur in biofilms (Steinberg et al., 2011). Biofilms on the surface of marine organisms are usually dominated by prokaryotes (Bacteria), while eukaryotes such as diatoms, fungi, and protozoa can be present at lower abundance (Bodammer and Sawyer, 1981; Höller et al., 2000; Burja and Hill, 2001; Hentschel et al., 2003; Webster and Taylor, 2012). Usually, the ratio bacteria:diatoms:flagellates in biofilms is 640:4:1 (Railkin, 2004). On undefended surfaces in temporal waters, bacterial densities typically reach densities of 10^7^ cm^−2^ or higher within a couple of weeks (Railkin, 2004 and references therein, Jones et al., 2008). The densities of epibiotic bacteria can vary substantially, depending on the species and their physiological status of the host but are typically lower. While the surfaces of some crustaceans such as the decorator crabs are heavily colonized (Hultgren and Stachowicz, 2011), surfaces of colonial didemnid tunicates remain almost free from microbes (Wahl and Lafargue, 1990). The abundance of the epibiotic bacterium *Pseudoalteromonas tunicata* on marine eukaryotic hosts is 3–4 orders of magnitude lower than on inert substrata (<1 × 10^3^ cells cm^−2^: Skovhus et al., 2004). Similarly, the densities of bacteria on soft corals were found to be low (about 5 to 10 × 10^3^ cells cm^−2^; Harder et al., 2003), cell densities which were similar to those on the alga *Caulerpa racemosa* (about 20 × 10^3^ cells cm^−2^; Dobretsov et al., 2006a). Densities of bacteria on the surface of the alga *Ulva reticulata* also were 2.3-fold lower than on undefended glass surfaces (about 27 × 10^3^ cells cm^−2^; Dobretsov and Qian, 2002). Densities of bacteria on the sponge *Haliclona* sp. were twofolds higher than on neighboring inanimate substrata, while those on the sponges *H. cymaeformis* and *Callyspongia* sp. were significantly lower (about 13 to 20 × 10^3^ cells cm^−2^; Dobretsov et al., 2005). The macroalga *Laminaria hyperborea* shows very variable cell densities in its biofilm (8.3 × 10^2^ to 6.3 × 10^7^ cm^2^: Bengtsson et al., 2010) while *Fucus vesiculosus* overall exhibits a more dense biofilm (7.7 × 10^6^ to 1.9 × 10^8^: Wahl et al., 2010).

**Table 1:** **Phylogenetic studies of the bacterial communities associated with macroalgae**.

Algal species	Molecular technique	Bacterial phyla	Country origin	Reference
**CHLOROPHYTA**				
*Bryopsis hypnoides*	CLO, FISH	BA, FI, PR (al, ep, ga)	Mexico	Hollants et al. (2011a,b)
*Bryopsis pennata*	CLO, FISH	BA, FI, PR (al, ep, ga)	Mexico	Hollants et al. (2011a,b)
*Caulerpa taxifolia*	CLO, RFLP	BA, **PR** (**al**, be, de, ga)	4 Countries	Meusnier et al. (2001)
*Chara aspera*	FISH	AC, **BA**, PL, **PR** (**al**, be, ga)	Germany	Hempel et al. (2008)
*Desmidium grevillii*	CLO	BA, PR (al, be, ga)	USA	Fisher et al. (1998)
*Halimeda opuntia*	CLO	AC, BA, CH, CL, CY, FI, PL, PR	N. Antilles	Barott et al. (2011)
*Hyalotheca dissiliens*	CLO	BA, PR (al, be, ga)	USA	Fisher et al. (1998)
*Spondylosium pulchrum*	CLO	BA, PR (al, be, ga)	USA	Fisher et al. (1998)
*Ulva australis*	CLO, DGGE	AC, **BA**, PL, **PR** (al, de, **ga**)	Australia	Longford et al. (2007)
*Ulva australis*	CLO	AC, **BA**, CY, PL, **PR** (**al**, de, ga), VE	Australia	Burke et al. (2011b)
*Ulva australis*	CFISH, DGGE	BA, **PR** (**al**, ga)	Australia	Tujula et al. (2010)
*Ulva intestinalis*	CLO, DGGE	AC, BA, **PR** (**al**, de, ep, ga), VE	Germany	Lachnit et al. (2011)
*Ulva prolifera*	CLO, DGGE	AC, BA, CY, FI, FU, PL, **PR** (**al**, be, de, ep, **ga**), SP, VE	China	Liu et al. (2011)
**HETEROKONTOPHYTA**				
*Dictyota bartayresiana*	CLO	AC, BA, CH, CL, CY, FI, PL, PR	N. Antilles	Barott et al. (2011)
*Fucus vesiculosus*	CLO, DGGE	**BA**, **CY**, PL, **PR** (**al**, be, de, ep, **ga**), **VE**	Germany	Lachnit et al. (2011)
*Laminaria hyperborea*	DGGE, FISH	AC, BA, CY, **PL**, **PR** (**al**, be, ga), VE	Norway	Bengtsson et al. (2010)
*Laminaria rodriguezii*	CLO	AR*, PR (be)	Spain	Trias et al. (2012)
*Saccharina latissima*	CLO, DGGE	BA, **PR** (**al**, **ga**)	Germany	Staufenberger et al. (2008)
**RHODOPHYTA**				
Coralline crustose	CLO	AC, BA, CH, CL, CY, FI, PL, PR	N. Antilles	Barott et al. (2011)
*Delisea pulchra*	CLO, DGGE	AC, BA, CH, CY, PL, **PR** (**al**, de, **ga**), VE	Australia	Longford et al. (2007)
*Delisea pulchra*	CLO, DGGE	BA, PL, **PR** (**al**, ga)	Australia	Fernandes (2011)
*Gracilaria vermiculophylla*	CLO, DGGE	AC, CY, DT, PL, **PR** (**al**, be, de)	Germany	Lachnit et al. (2011)
*Osmundaria volubilis*	CLO	AR*, PR (be)	Spain	Trias et al. (2012)
*Phyllophora crispa*	CLO	AR*, PR (be)	Spain	Trias et al. (2012)
*Porphyra yezoensis*	CLO	**BA**, LE, **PR** (al, be, **ga**)	Japan	Namba et al. (2010)
3 spp. macroalgae	CLO, TRFLP	**BA**, CY, PL, PR (al, ga), VE	Chile	Hengst et al. (2010)
12 spp. macroalgae	CLO	PL	Portugal	Lage and Bondoso (2011)
Unidentified turf algae	CLO	AC, BA, CH, CL, CY, FI, PL, PR	N. Antilles	Barott et al. (2011)

*The techniques utilized by the different authors for analyzing the microbial communities of brown (Heterokontophyta), green (Chlorophyta), and red (Rhodophyta) macroalgae are denaturing gradient gel electrophoresis (DGGE), fluorescence *in situ* hybridization (FISH), sequencing of 16S rRNA gene libraries (CLO), and terminal restriction fragment length polymorphism (TRFLP)*.

The bacterial phyla are represented by Actinobacteria (AC), Bacteroidetes (BA), Chlorobi (CL), Chloroflexi (CH), Cyanobacteria (CY), Deinococcus-Thermus (DT), Firmicutes (FI), Fusobacteria (FU), Lentisphaerae (LE), Planctomycetes (PL), Proteobacteria (PR) from which belong the bacterial classes Alpha-proteobacteria (al), Betaproteobacteria (be), Delta-proteobacteria (de), Epsilon-proteobacteria (ep) and Gammaproteobacteria (ga), Spirochaetes (SP), and the phylum Verrucomicrobia (VE). Also members of the Archaea (AR*) are considered. In bold are represented the dominant groups (when quantified).

Severe reduction of biofilm density relative to undefended surfaces and a specificity of their taxonomic composition (as treated below) indicate an active (pro-, antifouling) or passive (surface properties, exudates) role of the hosts in the recruitment of epibiotic bacteria.

### Biofilms on algae

Algae are a phylogenetically and morphologically extremely diverse group. They can be uni- to multicellular and from few μm to many m long. Although unicellular microalgae are subject to bacterial settlement (e.g., Grossart, 2010), structured microbial communities such as multispecies 3-D biofilms rarely develop on their surfaces (for references, see Follows and Dutkiewicz, 2011). This contrasts with multicellular (“macro-”) algae, which are especially susceptible to epibiosis and are typically covered by diverse microbial communities which may include bacteria, microalgae, fungi, and protists (Lobban and Harrison, 2000; Kohlmeyer and Volkmann-Kohlmeyer, 2003). Bacteria, typically by far the most abundant epibionts (see above), play a key role in the colonization and biofouling processes on macroalgae (Corre and Prieur, 1990): Algal tissue represents a rich source of organic nutrients which are a cue for some bacteria (e.g., Grossart, 2010). Since bacteria are omnipresent in the water column year-round, have a small reaction time, are highly adaptive and capable of rapid metabolization of algal exudates they are likely to be early colonizers (Fernandes, 2011), starting the biofilm process (e.g., Wahl, 1989; Goecke et al., 2010).

There is growing evidence that the composition of bacterial communities on the surface of macroalgae differs from that in the surrounding seawater or on inanimate (and undefended) substrata in close vicinity (e.g., Dobretsov et al., 2006a; Staufenberger et al., 2008; Lachnit et al., 2009; Bengtsson et al., 2010; Burke et al., 2011b). Comprehensive phylogenetic assessments of whole bacterial communities on algal surfaces are still scarce (but see Burke et al., 2011b). However, data of molecular studies (Table 1) – supported by culture-based studies (reviewed by Goecke et al., 2010) – are emerging and begin to provide important insights into the dynamic associations between macroalgae and bacteria. 16S rRNA gene sequences retrieved from epiphytic bacteria on freshwater and marine macroalgae belong to several major lineages within bacteria: Alpha, Beta, and Gamma classes of the Proteobacteria, the Bacteroidetes, and the Actinobacteria (Fisher et al., 1998; Longford et al., 2007; Hempel et al., 2008, see Table 1). At a higher taxonomic level, the microbial groups that dominate surface communities on macroalgae – Proteobacteria and Bacteroidetes – are the same as in most aquatic environments (Cottrell and Kirchman, 2000; Sapp et al., 2007). A prevalence of sequences from these two bacterial phyla has been detected in phytoplankton (Riemann et al., 2000; Schäfer et al., 2002), and on green algae (Meusnier et al., 2001; Longford et al., 2007; Hempel et al., 2008; Burke et al., 2011b; Hollants et al., 2011a; Liu et al., 2011), brown algae (Staufenberger et al., 2008; Wiese et al., 2009; Bengtsson et al., 2010; Lachnit et al., 2011), and red algae (Namba et al., 2010; Fernandes, 2011; Lachnit et al., 2011), but also on invertebrates (Tait et al., 2007; Mangano et al., 2009, among others), suggesting that those marine bacteria are common and – possibly – important micro-epibionts on many different organisms. Especially the *Roseobacter* clade of the Alpha-proteobacteria has been identified as one of the most prevalent groups in the bacterial assemblages associated with phytoplankton (Schäfer et al., 2002; Seyedsayamdost et al., 2011) and macroalgae from different geographical locations (Staufenberger et al., 2008; Hengst et al., 2010; Namba et al., 2010; Tujula et al., 2010; Fernandes, 2011; Liu et al., 2011). Other phyla, such as the Planctomycetes, Verrucomicrobia, and Cyanobacteria (Bengtsson et al., 2010; Lachnit et al., 2011; Lage and Bondoso, 2011) have just recently been recognized as frequent colonizers of macroalgal surfaces. Members of the Firmicutes are frequently found among the cultivatable bacteria associated with macroalgae and are usually also relatively prominent among the total bacteria that are identified in molecular studies (Wiese et al., 2009; Goecke et al., 2010). Other phyla detected less frequently on the surfaces of macroalgae are Chlorobi, Chloroflexi, Deinococcus-Thermus, Delta-proteobacteria and Epsilon-proteobacteria, Fusobacteria, Lentisphaerae, and Spirochaetes (but, see Barott et al., 2011; Liu et al., 2011, Table 1). Furthermore, members of the domain Archaea have been very recently detected on macroalgae in mesophotic depth in Spain (Trias et al., 2012).

This high diversity of bacterial epibionts is not randomly distributed among algal host species. More recent research confirms that different species of marine macroalgae in the same habitat support differently composed bacterial communities (Lachnit et al., 2009, 2011; Nylund et al., 2010; Trias et al., 2012), while specimens of the same algal even in different environments tend to be associated with highly similar bacterial communities (Staufenberger et al., 2008; Lachnit et al., 2009). The relationship between environmental factors and non-epibiotic bacterial abundance and community composition has been well documented in various marine ecosystems (Sapp et al., 2007). Even on two conspecific host individuals a complete overlap in the epibiotic microbial communities cannot be expected, because aquatic systems are usually subject to drastic spatial, temporal (seasonal) and post-disturbance shifts (Corre and Prieur, 1990; Longford et al., 2007; Staufenberger et al., 2008; Fernandes, 2011; Liu et al., 2011), and the physiological state of the host (age, senescence, diseases) may affect the associated bacterial community via exuded metabolites (Goecke et al., 2010; Seyedsayamdost et al., 2011).

### Biofilms on animals

Recent studies suggested that surfaces of most invertebrates and vertebrates stay relatively free from macrofouling while they usually feature some degree of microbial fouling (Richmond and Seed, 1991; Dobretsov et al., 2006b). Both, culture dependent and polymerase chain reaction (PCR) based studies have revealed that microorganisms associated with animals differ from those in the water column and those associated with other types of substrata in the neighborhood, suggesting that these associations are specific to some degree (Burja and Hill, 2001; Harder et al., 2003; Hentschel et al., 2003; Lee and Qian, 2003; Thakur et al., 2004; Qian et al., 2006; Webster and Taylor, 2012). Most of these studies are based on investigation of sponge – associated endosymbiotic microorganisms, while the information about microbes internally or externally associated with other animals is limited (but, see: Bodammer and Sawyer, 1981; Pukall et al., 2001; Harder et al., 2003; Kittelmann and Harder, 2005; Perez-Matos et al., 2007; Winters et al., 2010). It has been demonstrated that the community composition of epibiotic bacteria associated with the same sponge species from different locations remained consistent (Lee et al., 2006a, 2011), while microbial communities associated with different species of sponges differed substantially (Qian et al., 2006; Lee et al., 2011). This suggests a certain host-specificity of the biofilms (as it has been shown in algae) while in the vast majority of cases a mandatory restriction of a given bacterial strain to a particular host species has not yet been shown.

The composition of epibiotic bacterial communities associated with marine organisms is influenced by temporal changes in the environment (Thakur et al., 2004; Lee et al., 2006b). However, some particular bacteria are specifically and persistently associated with particular marine animals and not present in seawater or on other animals (Thakur et al., 2004; Sharp et al., 2007). For example, *Candidatus Endobugula sertula* is specifically associated with the surface of bryozoan larvae *Bugula neritina* and protects them from predatory fishes (Sharp et al., 2007). Another bacterium – *Bacillus* sp. – was always and exclusively associated with surfaces of the sponge *Ircinia fusca* (Thakur et al., 2004). Besides few cases (see Gustafson and Reid, 1988), it is uncertain whether specific animal symbionts are transmitted vertically via gametes or larvae from adults. The study by Sharp et al. (2007) demonstrated that the mass spawning corals *Montastraea annularis*, *M. franksi*, *M. faveolata*, *Acropora palmata*, *A. cervicornis*, *Diploria strigosa*, and *A. humilis* do not transmit their epibiotic bacteria via their gametes, and bacteria colonize corals only after their settlement and metamorphosis. This suggests that interactions between juvenile forms and epibiotic bacteria are particularly important for the formation of host-specific assemblages of bacteria.

The density of epibiotic bacteria on animal surfaces varies enormously at numerous scales from within-individual to among species, habitats, regions, and seasons (see references cited in the previous paragraph). Some didemnid ascidians exhibit an almost sterile surface with 0 to 1.5 × 10^2^ cells cm^−2^ (Wahl and Lafargue, 1990). Epibacterial densities on sponge surfaces range from almost sterile (60 cells cm^2^: *Crambe* crambe,), over strongly reduced (3 to 4 × 10^4^ cells cm^−2^: *Ircinia fasciculata*, *Spongia officinalis*, Becerro et al., 1994) to “normally fouled” (6.93 × 10^6^ cells cm^2^: *Ceratoporella nicholsoni*, Santavy et al., 1990; 7 to 15 × 10^6^ *Ircinia ramosa*, Thakur and Anil, 2000). Corals may have low (5 × 10^5^ cells cm^−2^: various species, Koh, 1997) or remarkably high densities of epibiotic bacteria (8.3 × 10^8^ cells cm^−2^: *Oculina patagonica*; Koren and Rosenberg, 2006). The bacterial densities on the carapaces of a variety of crustaceans ranged between 7 × 10^4^ and 3 × 10^6^ cells cm^−2^, Becker, 1996). The bryozoan *Conopeum reticulatum* features 5 × 10^7^ cells cm^−2^ on its surface (Kittelmann and Harder, 2005). When the bacterial density in the epibiotic biofilm is substantially reduced relative to neighboring species or inanimate substrate in the habitat, the host surface apparently is unsuitable for settlement and/or growth of bacteria due either to physiological exchange processes through the epidermis (e.g., extreme pH fluctuations during diurnal switches between photosynthesis and respiration) or to the deployment of defensive secondary metabolites. However, antimicrofouling mechanisms of the host are not subject of this review.

### Internal associations

Although not the prime focus of this review, internal association will be briefly treated here because they frequently derive from epibiotic biofilms. Certain types of bacteria have been able to penetrate the host tissue and even overcome the cell membrane and develop an obligatory dependence between bacteria and host (see Woyke et al., 2006; Thornhill et al., 2008; Hollants et al., 2011a). Such endosymbioses with prokaryotes have been established multiple times in many of the major metazoan groups and the diversity of these associations demonstrates their plasticity and evolutionary success (Dubilier et al., 1999; McFall-Ngai, 2000). This is not surprising because many symbionts have an important, mostly beneficial effect on their host, although pathogenic and saprophytic relationships are also involved (Sipe et al., 2000; Woyke et al., 2006; Goecke et al., 2010). The transmission of endosymbionts proceeds in one of three ways: By vertical transmission (transfer from parent to offspring), by horizontal transmission (involving the spread of symbionts between neighboring hosts), or by reinfection of the new host generation from the environmental stock of microorganisms (see Gustafson and Reid, 1988). The bacterial symbiont is not a passive player in the colonization process (McFall-Ngai, 2000). Both, the horizontal transmission of endosymbionts or the reinfection of the new host generation from an environmental stock of microorganisms are likely to involve contact with the host’s biofilm. This was proven for, e.g., *Vibrio fischeri* which colonizes the light organs of squid only after specific contact based on both, host ciliar structures and bacterial cell wall components to the juveniles (Visick and Ruby, 2006). Unfortunately, the cultivation of those microbial consortia in the absence of their host is hindered by severe technical difficulties (McFall-Ngai, 2000), which are a barrier to the further elucidation of their biological roles (Moss et al., 2003) and competition during the recolonization of the host’s offspring.

## Ecological Role of Epibiotic Bacteria: Modulation of Host-Environment Interactions

The recent increase in studies of the phylogenetic diversity of bacterial communities associated with marine organisms starts to provide information on the presence and absence of specific taxa under various environmental conditions and on different hosts. However, it provides little information on the ecological function of these taxa. The *in situ* functioning of epibiotic strains or communities is difficult to study. A new and promising approach is metagenomic sequence analysis, which was used to investigate the relation between community structure and community function in the bacterial assemblages associated with *Ulva australis* (Burke et al., 2011b). Despite a high phylogenetic variability in the microbial species composition the authors discovered only little functional variability (measured as presence of functional gene clusters). Phylogenetically different bacterial species (or strains) of the regional/seasonal colonizer pool – able to colonize one particular host species – that can carry out similar metabolic and other functions apparently compete with each other in the colonization of algal surfaces (Burke et al., 2011a; Fernandes, 2011). Due to remarkable functional redundancy structural differences in the epibiotic biofilm are not necessarily associated with a shift in function. Since for the host and its interactions with the environment biofilm function matters more than phylogenetic biofilm composition, investigations at the functional level based on genomic or metabolomic information should become more prominent in the future.

The composition and metabolism of a biofilm have the capacity to substantially modulate the interactions of the host with its living and non-living environment (see below). Both traits of the biofilm are affected by host properties (not treated here), environmental conditions and interactions within the biofilm. The complex architecture of a mature biofilm provides niches with distinct physico-chemical conditions, differing, e.g., in oxygen availability, in concentration of diffusible substrates and metabolic side products, in pH, and in the cell density (Costerton et al., 1999). In such a mixed microbial community the strains may interact antagonistically or synergistically with each other, the latter resulting in co-colonization of distinct groups of bacteria having metabolic cooperation (Kuchma and O’Toole, 2000; Andersson et al., 2008; Nadell et al., 2009). Microbial processes such as nitrification, anaerobic degradation of organic compounds, or bioremediation of xenobiotic compounds, have been shown to require interactions between different bacterial species within the biofilm (Paerl and Pinckney, 1996). This metabolic cooperation is advantageous to the micro-community. Nevertheless, cooperation among species is only expected under restricted conditions (Nadell et al., 2009). Under natural conditions, bacteria compete (intra- or inter-specifically) intensely with their neighbors for space and resources. A surface (especially of hosts) may itself also be a trophic source where attached microorganisms catabolize organic or inorganic nutrients directly (Madigan and Martinko, 2006; Grossart, 2010). Therefore, the presence of other microorganisms on a surface reduces the availability of substrate and substratum for colonizing species (Prado and Kerr, 2008).

Under such competitive selection it is not surprising that bacteria have developed special mechanisms in order to interfere with the capability of other antagonistic bacteria during the process of surface colonization and acquisition of nutrients (Falagas et al., 2008). The mechanisms of bacterial antagonism would include depletion of some essential substances (e.g., a substrate or a vitamin), alteration in the microenvironment (e.g., changes in the gas concentration or pH), or production of an antagonistic substance (e.g., antibiotics; Wannamaker, 1980), but also the presentation of a real obstacle or barrier to other microorganisms by competing directly for the host-cell-binding sites (as shown by Reid et al., 2001). Clearing a space to colonize by eliminating prior residents can be accomplished by production of antimicrobials or by production of molecules that facilitate the competitor’s dispersal without actually killing them (see Modulation of Bacterial Settlement by Epibiotic Bacteria and Quorum Sensing and its Modulation below, Hibbing et al., 2010).

Each specific biofilm by its physical structure, its functional components, and their metabolic activity will affect host interactions differently. In the following we will concentrate on the aspects (i) how biofilms by physical insulation and metabolic filtration affect the host’s access to matter and energy and (ii) how biofilms – mainly due to released infochemicals – modulate the interactions between host and further colonizers, potential consumers, and – very summarily – pathogens.

### Modulation of the access of the host to resources (nutrients, gases, light, infochemicals, toxins)

Epibiotic biofilms constitute a physical and physiological barrier between their host and the environment. How biofilms at different stages of their development interfere with their substrate’s surface properties in general and transfer of matter and energy through the fouled surface in particular has been investigated at great length for technical surfaces, such as reverse osmosis membranes in desalination plants, submerged optical, and other sensors or heat exchange devices (e.g., Winters and Isquith, 1979; Flemming, 1997; Baker and Dudley, 1998; Kerr and Cowling, 1998). Presumably, the passage of chemicals and radiation across these membranes is modulated by microfouling quite analogously to what is happening at the living surfaces of marine organisms covered by epibiotic biofilms. However, this insulating or filtering function of biofilms is much less studied in epibiotic associations because typically these biofilms cannot be maintained structurally and functionally intact in the absence of the host. Based on the few studies available (references in Wahl, 2008a) and extrapolated from the more technical studies mentioned before the following effects of epibiotic bacterial biofilms on their hosts have been shown or are plausible.

Physically, the biofilms represent the new functional interface between the host and environment replacing many properties of the host’s surface, such as color, microtexture, or wettability by the corresponding biofilm properties (e.g., Becker and Wahl, 1991; Bers et al., 2006). Irradiation of optical sensors (eyes or more primitive photoreceptors) and of photosynthetic organelles (chloroplasts) may be reduced by the presence of biofilms (e.g., Philip-Chandy et al., 2000; Head et al., 2004). Bacterial biofilms only few weeks old may reduce the incoming light by over 50% (Wahl et al., 2010), which undoubtedly would severely affect the photosynthetic performance of primary producers and, consequently, their depth distribution. This investigation also highlighted that warming accelerates the formation of shading biofilms, putting a greater challenge on protective measures by the host. Without antimicrofouling defenses, at 25°C naturally establishing biofilms absorb 95% of the incoming light, virtually blinding fouled photoreceptors. It is likely, but unproven, that the diffusion of gases (CO_2_, O_2_) through the host’s epidermis is compromised by biofilms. Thus, the metabolism of primarily heterotrophic biofilms will deplete O_2_ and enrich CO_2_ before they reach the host surface. Similarly, the access of the host to nutrients in the water column (nitrate, phosphate, bicarbonate, micronutrients, vitamins, amino acids, polycarbonates, etc.) can be hindered by reduced diffusion through or pre-emption by a biofilm (as suggested by numerous studies on the role of biofilm in water purification, e.g., Terada et al., 2006). In contrast, some nutrients including vitamins or growth factors are provided to the host by epibiotic biofilms (e.g., Chisholm et al., 1996; Seyedsayamdost et al., 2011). In certain extreme environments (as cold seeps and black smokers) biofilms may constitute the trophic interface enabling the host to live on otherwise toxic compounds (e.g., Goffredi, 2010). Epibiotic microorganisms may interfere with the reception or release of infochemicals which serve communication between conspecifics or between interacting species (see below Modulation of Eukaryote Settlement by Epibiotic Bacteria). All these insulating (or degrading) effects of the biofilm can be beneficial when the factors warded off are potentially harmful to the host such as UVR, toxins or infochemicals used by searching foes (consumers, parasites, pathogens; e.g., Steinberg et al., 2011). Also, some larvae use the chemical cues emitted by characteristic biofilms on adult conspecifics for gregarious settlement (De Gregoris et al., 2012).

### Fouling modulation by epibiotic biofilms

#### Surface modification

The characteristics of the substratum have a significant effect on the rate and extent of attachment of microorganisms (Donlan, 2001). Surface roughness and microtopographical features have been postulated as one aspect of mechanical antifouling defense mechanisms of some invertebrates, for example, by the development of small spicule-like or ripple-shaped structures (Bers and Wahl, 2004). Wettability of surfaces also affects, and in certain ranges hinders, attachment (e.g., Becker et al., 2000). This notwithstanding, colonizing organisms, and in particular bacteria, have evolved many mechanisms that allow them to colonize a host surface (Reid et al., 2001) and to form a biofilm on it. Those biofilms confer special properties to the surface of the substratum that may completely mask the properties, including the physical fouling-reducing surface properties just mentioned, of the underlying substratum itself (Donlan, 2001; Bers et al., 2006). Biofilm surfaces vary from smooth and confluent to rough and uneven with tall cell clusters interweaved by fluid-filled channels (see Nadell et al., 2009). Surface modifications by epibiotic biofilms comprise the alteration of the surface chemical composition and morphology, surface topography and roughness, the hydrophilic/hydrophobic balance, as well as the surface energy and polarity (Vladkova, 2009). The progressive recruitment of micro-colonizers and their production of a mucus extracellular polysaccharide matrix gradually covers the host’s features which may facilitate settlement of macro-colonizers that previously discriminated against host’s features (Costerton et al., 1995; Bers and Wahl, 2004; Vladkova, 2009). Mature biofilms will to some degree control the flux of energy and matter through the host’s body surface (e.g., Costerton et al., 1987; Dobretsov et al., 2006b; Wahl, 2008b), altering the chemical properties of the boundary layer.

Biofilms comprise not only cells but also a myriad of compounds that these cells release into the biofilm matrix and the boundary layer (Nadell et al., 2009). By overlaying host attributes by its own chemical information a biofilm may promote further colonization by some or deter colonization by other potential foulers (see Joint et al., 2000; Dobretsov, 2009).

#### Modulation of bacterial settlement by epibiotic bacteria

Antagonism plays a significant role in shaping bacterial communities (Mangano et al., 2009). The production by microbes of secondary metabolites against potential competitors, predators, or antagonists may indirectly affect the host and its biological interactions with foulers and pathogens (e.g., Gil-Turnes et al., 1989; Armstrong et al., 2001; Steinberg et al., 2011). Space and nutrient limitation are enforcing surface dwelling microorganisms to evolve particular adaptive responses to prevent colonization or growth of potential competitors (Egan et al., 2008). From an ecological point of view, inhibitory interactions among bacteria inhabiting the same niche represent an interesting evolutionary strategy, conferring a selective advantage in competition, and acting as an effective control of microbial populations (Hentschel et al., 2001). In some cases, one organism may inhibit growth or metabolism of other organisms directly by excretion of a specific inhibitor (Wannamaker, 1980). In other cases the effect is indirectly or at least non-specifically mediated by the physiological activities of the organism producing, e.g., acids from the fermentation of sugars (Madigan and Martinko, 2006). Such responses may include induction of negative chemotaxis in potentially competing bacteria or the mentioned interference with processes leading to the irreversible attachment of cells to substratum (Boyd et al., 1999). The specific mediators playing a role in a bacterial antagonism range from rather complex substances (such as bacteriocins and enzymes) to simple molecules (such as ammonia, lactic acid, free fatty acids, and hydrogen peroxide; Wannamaker, 1980).

Bacteria producing antimicrobial and other bioactive compounds have been isolated from a range of marine invertebrates and algae including ascidians, bryozoans, corals, crustaceans, mollusks, sponges, tubeworms, etc. (Table 2). They have yielded a large number of new natural products as arenimycin, bacillistatins, bogorol, harman, lutoside, salinamides, sesbanimides, among many others (Acebal et al., 1998; Bultel-Poncé et al., 1998; Moore et al., 1999; Barsby et al., 2002; Aassila et al., 2003; Pettit et al., 2009; Asolkar et al., 2010). It should be cautioned at this point, that in the majority of studies “bioactivities” are not assessed at natural *in situ* concentrations of the compounds. One reason is that in many cases the research motivation was pharmacological rather than ecological, another reason being that metabolite concentrations in the boundary layer are difficult to determine. In these cases, an extrapolation of the *in vitro* results to a real function within the biofilm *in vivo* is problematic (e.g., Clare, 1996). The production of antimicrobial compounds is not restricted to a certain bacterial group but instead appears to be wide spread across various bacterial phyla (Penesyan et al., 2009), and is neither limited to a geographical region or habitat (see Table 2). Additionally, a single microorganism has the potential to produce many different compounds under different conditions (Bode et al., 2002). It is thought that a generalist bacterial species occupying a broad spectrum of environments (i.e., *Bacillus*, *Pseudoalteromonas*, or *Streptomyces*) would be more likely to benefit from producing broad spectrum antimicrobials or a cocktail of toxins targeting different potential competitors, while those organisms highly specialized for a given habitat (i.e., obligate epiphytes) may produce antimicrobials with narrower range, targeting specific competitors (Hibbing et al., 2010). Although production of bioactive compounds is a characteristic feature of some bacteria and may largely promote the colonization of and competition on host surfaces (Holmström and Kjelleberg, 1999; Patel et al., 2003; Rao et al., 2005), they can be costly in terms of resource allocation, diverting energy away from growth and reproduction (Kumar et al., 2011). To effectively inhibit competitors, the antibiotic must be produced in sufficient quantity, and this may require the concerted effort of a population. Accordingly, antibiotic production often is regulated by a QS mechanism (Hibbing et al., 2010). Antimicrobials have been postulated to be (in nature) rather signaling molecules within species than chemical weapons (Hibbing et al., 2010). Supporting this view, antibiotics are often produced at sub-inhibitory concentrations, the metabolic costs of their production are relatively high, and many bacteria have a capacity for fast evolutionary development of tolerance against antimicrobials (Hibbing et al., 2010). The presumed predominance of an informational function of secondary metabolites has led to the emergence of a new field of research named neuroecology (e.g., Steinberg et al., 2011). Given the ability of bacteria to escape potentially harmful environments in response to sub-lethal concentrations of such chemo-effectors, the metabolites responsible for mediating antifouling mechanisms may well be overlooked using standard antimicrobial assays (Young and Mitchell, 1973). The abundance of behavioral (deterrent) effects relative to lethal (antibiotic) effects in defensive metabolites has been shown for marine invertebrates and algae (Wahl et al., 1994; Engel et al., 2002). With conventional testing these ecologically important activities would go undetected.

**Table 2:** **Antimicrobial activity of epibiotic bacterial strains isolated from different hosts**.

Host	Total strains	Active strains	% of active strains	Test	Country	Reference
**MACROALGAE**						
*Saccharina latissima*	210	103	50	lb	Germany	Wiese et al. (2009)
Invertebrates 4spp., alga	400	140	35	lb, env	Scotland	Burgess et al. (1999)
Macroalgae 5 spp.	224	38	16.9	lb	Spain	Lemos et al. (1985)
Macroalgae 7 spp.	280	60	21	env	Scotland	Boyd et al. (1999)
Brown algae 9 spp.	116	23	20	lb, env	Japan	Kanagasabhapathy et al. (2006)
Red algae 9 spp.	92	31	33	lb, env	Japan	Kanagasabhapathy et al. (2008)
Macroalgae 2 spp.	325	39	12	lb	Australia	Penesyan et al. (2009)
*Ulva lactuca*	10	6	60	env	Fiji	Kumar et al. (2011)
**INVERTEBRATE**						
*Acropora formosa*	354	36	10	lb	India	Chellaram et al. (2011)
*Anoxycalyx joubini*	38	–	90	env	Antarctica	Mangano et al. (2009)
*Balanus amphitrite*	28	4	14.3	lb	India	Jebasingh and Murugan (2011)
Bryozoa 14 spp.	340	101	29.7	lb	MS, BS	Heindl et al. (2010)
Coral 2 spp.	352	46	13	lb, env	India	Gnanambal et al. (2005)
Coral 9 spp.	78	19	24.3	lb, env	Israel	Shnit-Orland and Kushmaro (2009)
Echinoderms 2 spp.	9	9	100	lb	India	
*Favia palida*	335	41	13	lb	India	Chellaram et al. (2011)
*Haliclona simulans*	52	30	57.6	lb	Ireland	Kennedy et al. (2009)
*Haliclona* sp.	56	8	14.3	lb	Indonesia	Radjasa et al. (2007)
Invertebrates 14 spp.	105	14	13	lb, env	Australia	Wilson et al. (2011)
Invertebrates spp.	290	54	18.6	lb	Venezuela	Castillo et al. (2001)
*Lissodendoryx nobilis*	37	–	62.2	env	Antarctica	Mangano et al. (2009)
*Mycale adhaerens*	20	15	75	env	Hong Kong	Lee and Qian (2004)
*Penaeus monodon*	185	49	26.3	env	India	Shakila et al. (2006)
*Petrosia ficiformis*	57	5	8.7	lb, env	Italy	Chelossi et al. (2004)
*Sarcophyton* sp.	98	6	6.3	env	Indonesia	Sabdono and Radjasa (2006)
Sponge 2 spp.	238	27	11.3	lb	MS	Hentschel et al. (2001)
Sponge 4 spp.	28	4	14.3	lb	India	Nair et al. (2011)
Sponge 11 spp.	20	10	50	lb	MS	Abdelmohsen et al. (2010)
Sponge 9 spp.	158	12	7.6	lb	Brazil	Santos et al. (2010)
Sponge 10 spp.	2562	283	15.2	lb	MS	Muscholl-Silberhorn et al. (2008)
Sponge 4 spp.	75	16	21	lb	India	Anand et al. (2006)
Sponge 5 spp.	26	21	80.7	lb	India	Gandhimathi et al. (2008)
Sponge 4 spp.	94	58	61.7	lb	India	Dharmaraj and Sumantha (2009)

*Where lb, laboratory test strains, env, wild strains, BS, Baltic Sea, MS, Mediterranean Sea. Note that only exceptionally natural concentrations were known, and test concentrations may differ substantially from these*.

Bacteria producing antibiotic substances are more prevalent in epibiotic biofilms than in other habitats, such as seawater (Mearns-Spragg et al., 1998; Zheng et al., 2005; Kanagasabhapathy et al., 2008, see Table 2). Furthermore, some bacteria may actually produce active compounds only when they are cultivated on surfaces, as for example one strain of *Bacillus licheniformis*, which lost the bioactivity when it was cultured in a liquid medium instead of on agar (Yan and Boyd, 2002; Matz et al., 2008). It cannot be decided now whether this is a result of a better energy supply in the epibiotic microhabitat or of enhanced competition in the biofilm (Kanagasabhapathy et al., 2008; Mangano et al., 2009; Steinberg et al., 2011).

There is still a long way to go if we wish to understand what kind of compounds bacteria produce under which set of environmental parameters *in situ*. Their behavior may be affected by many factors (Bode et al., 2002) including chemical signal from the host (e.g., Dworjanyn and Wright, 2006; Steinberg et al., 2011), and it is difficult to know whether, when, or how micro-epibionts possibly protect their hosts. Bacteria frequently change their metabolic profile once they are outside of their natural habitat because of altered growth conditions and lack of selective pressure (Mangano et al., 2009). For example, a marine actinomycete (strain SS-228) was shown to produce an antibiotic compound only when the growth medium was supplemented with *Laminaria* sp., a macroalga common in the habitat from which the strain was obtained (Okazaki et al., 1975).

Also, strain specific variation of the production of antibiotics is a well documented phenomenon, e.g., in *Pseudoalteromonas* and *Bacillus* strains (see Todorova and Kozhuharova, 2010; Vynne et al., 2011) and this may have two reasons: First, bacteria quickly adapt to the environment and the production of secondary metabolites relies on many different factors (Bode et al., 2002). Second, plasmids, transposons, or phages may enable the mobilization and transfer of biosynthetic operons between different bacterial strains and even across the species barrier (Martin and Liras, 1989), which in combination with rapid growth rates and large population sizes results in the introduction of many unique mutations that even at low frequencies may rise to variants that are more adapted or biologically active (Nadell et al., 2009; Hibbing et al., 2010).

#### Modulation of eukaryote settlement by epibiotic bacteria

Biofilms may either enhance or inhibit settlement of propagules (reviewed by Dobretsov et al., 2006b; Qian et al., 2006; Prendergast et al., 2009; Hadfield, 2011, Table 3). Since the response to a biofilm differs among potential settlers, the influence of a biofilm on recruitment also has a selective aspect. All components of marine biofilms (bacteria, diatoms, fungi, and protozoa) may potentially affect larval and algal settlement through physical modification of surfaces and production and release of molecular cues or deterrents.

**Table 3:** **Inhibition of larval settlement by epibiotic bacteria and compounds from these, ?–no data available**.

Host	Bacteria	Effective against	Active compound	Reference
Barnacle *B. amphitrite*	*Vibrio*, *Alteromonas*, *Alcaligenes*, *Flavobacterium*, and *Pseudomonas*	Barnacle *B. amphitrite*	?	Mary et al. (1993)
Algae, sponges, and ascidian *Ciona intestinalis*	*Pseudomonas aurantia*, *P. citrea*, *P. piscicida*, *P. rubra*, *P. undina*, *P. ulvae*, *P. haloplanktis*, *P. luteoviolacea*, *P. tunicata*, *P. nigrifaciens*	*Polychaeta, Hydroides elegans*, barnacle *Balanus amphitrite*, and ascidian *Cliona intestinalis*	?	Holmström and Kjelleberg (1999), Holmström et al. (2002)
Nudibranch *Archidoris pseudoargus*	*Pseudomonas* sp.	Barnacle *Balanus amphitrite*	Phenazine-1-carboxylic acid, 2-n-hyptyl quinol-4-one, 1-hydroxyphenazine phenazine-1-carboxylic acid, pyolipic acid	Burgess et al. (2003)
Green alga *Ulva reticulata*	*Vibrio alginolyticus*	*Polychaeta, Hydroides elegans*	Polysaccharide >200 kDa consist of glucose, mannose, galactose, and glucosamine	Dobretsov and Qian (2002), Harder et al. (2004)
Soft coral *Dendronephthya* sp.	*Vibrio* sp., uncultured *Ruegeria*, unidentified *a*-*Proteobacterium*	*Polychaeta, Hydroides elegans*, bryozoan *Bugula neritina*	?	Dobretsov and Qian (2004)
Sponge *Halichondria okadai*	*Alteromonas* sp.	Barnacle *B. amphitrite*	Ubiquinone	Kon-ya et al. (1995)
Ascidian *Stomozoa murrayi*	*Acinetobacter* sp.	Barnacle *B. amphitrite*	6-bromindole- 3-carbaldehyde	Olguin-Uribe et al. (1997)
Green alga *Ulva australis*	*Pseudoalteromonas tunicata* and *Phaeobacter* sp. strain 2.10	Bryozoan *Bugula neritina*	?	Rao et al. (2007)
Brown alga *Fucus serratus*, *F. vesiculosus*, and the red alga *Polysiphonia stricta*	Natural communities, *Photobacterium halotolerans* and *Ulvibacter litoralis*, *Shewanella basaltis*, *Pseudoalteromonas arctica*, *Shewanella baltica*, and *Bacillus foraminis*	Barnacle *Amphibalanus improvisus*	?	Nasrolahi et al. (2012)
Sponge *Lissodendoryx isodictyalis*	*Winogradskyella poriferorum*	Polychaete *H. elegans* and barnacle *B. amphitrite*	Poly-ether AE	Dash et al. (2009), Dash et al. (2011)

Several independent studies have shown that strains of epibiotic bacteria associated with sponges (Lee and Qian, 2003), soft corals (Dobretsov and Qian, 2004), tunicates (Szewzyk et al., 1991; Egan et al., 2008), and algae (Dobretsov and Qian, 2002) can be grouped into three functional groups based on their bioactivity toward a given macrofouler. They can be inductive (induce settlement), non-inductive or neutral (do not induce settlement), and inhibitive (significantly reduce larval settlement).

Almost all (epibiotic) biofilms in the sea are multispecies (Wieczorek and Todd, 1998; Dobretsov, 2010). Their effects may differ from those of monospecific bacterial biofilms (Tran and Hadfield, 2011), which possibly explains the fact that most studies of natural assemblages so far only reported inhibitory effects (references within Dobretsov et al., 2006b and this review). For example, artificial biofilms composed of 11 “inductive,” “neutral,” and “inhibitive” strains from the soft coral *Dendronephthya* sp. at a 1:1:1 ratio inhibited larval settlement of *Hydroides elegans* and *B. neritina* in a laboratory experiment (Dobretsov and Qian, 2004, Table 3). The bioactivity of multispecies biofilms depends not only on the presence of particular bacterial taxa but also on their proportional abundance (Lau and Qian, 1997; Harder et al., 2002; Dahms et al., 2004). Further, bacteria potentially produce different types or quantities of settlement modulating compounds under laboratory conditions and in the field, as the biotic and abiotic environment usually determines bacterial behavior (reviewed by Dobretsov et al., 2006b). Below we will provide some examples of epibiotic bacteria that induce or inhibit the settlement of potential eukaryote foulers through a release of bioactive compounds. It should be noted at this point, that this infochemical (or “neuroecological”) interaction is bidirectional: numerous propagules cue on biofilm signals to detect suitable habitats or reject unsuitable ones (e.g., Hadfield, 2011) and it is difficult to differentiate this behavior from a “repellent” or “attractant” activity of a bacterial metabolite. In most cases, these aspects are probably the two sides of the same coin.

Numerous examples demonstrate that epibiotic biofilms induce larval settlement of cnidarian, mollusks, and polychaete species (reviewed by Wieczorek and Todd, 1998; Prendergast et al., 2008). In a laboratory experiment, biofilms from the green filamentous alga *Cladophora rupestris* attracted larvae of *Mytilus edulis*, while biofilms from the brown alga *Laminaria saccharina* repelled them (Dobretsov, 1999). *Macrococcus* sp. AMGM1 and *Bacillus* sp. AMGB1 isolated from the surfaces of marine seaweeds and mussels significantly increased larval settlement of *Perna canaliculus* (Ganesan et al., 2010). Bacteria belonging to the genera *Vibrio* and *Pseudoalteromonas* associated with the shells of *B. amphitrite* induced gregarious settlement of the host species (De Gregoris et al., 2012). In this study, even small variations in the proportion of the species of the biofilms produced different effects on larval settlement. While the phenomenon of settlement induction driven by epibiotic bacteria is widespread (Dobretsov, 2009), to date only few inductive compounds from epibiotic bacteria have been isolated. This includes tetrabromopyrrole that induced larval attachment and metamorphosis of the acroporid coral larvae *Acropora millepora*, and was produced by *Pseudoalteromonas* strains associated with the crustose coralline algae (Tebben and Tapiolas, 2011).

It has been previously proposed that mainly *Pseudoalteromonas* species inhibit settlement of propagules (Holmström and Kjelleberg, 1999; Holmström et al., 2002). Nowadays, we know that there is no correlation between the inhibition of larval settlement and bacterial phylogeny (reviewed by Dobretsov et al., 2006b, Table 3). In a pioneer work on bacteria associated with the shells of the barnacle *B. amphitrite*, Mary et al. (1993) demonstrated that 12 out of 16 isolates inhibited larval settlement. In another study, the activity of 10 *Pseudoalteromonas* species isolated from marine sponges, algae, and tunicates on the settlement of larvae of the barnacle *B. amphitrite* and the polychaete *H. elegans* has been investigated in laboratory experiments (Holmström et al., 2002). Only *P. tunicata*, *P. citrea*, and *P. ulvae* inhibited settlement of both larval species and the bacterium *P. tunicata* was the best performing bacterium. Surprisingly, *P. tunicata* isolated from Baltic macroalgae did not inhibit settlement of *Amphibalanus improvisus* larvae (Nasrolahi et al., 2012). In contrast, monospecific bacterial films of *Shewanella baltica* and *Pseudoalteromonas arctica* associated with the red alga *Polysiphonia stricta*, *Photobacterium halotolerans*, and *Ulvibacter litoralis* isolated from *F. serratus*, and *S. baltica* and *Bacillus foraminis* isolated from *F. vesiculosus* reduced the attachment of cyprids of *A. improvisus* (Nasrolahi et al., 2012). The strongest inhibitory effect was obtained with isolates from *P. stricta*. Biofilms and conditioned seawater from seven isolates obtained from the alga *U. reticulata* reduced settlement of *H. elegans* larvae (Dobretsov and Qian, 2002) and the antifouling compound from the epibiotic *Vibrio* sp.2 (identified later as *V. alginolyticus*) was identified as a large >200 kDa polysaccharide consisting of glucose, mannose, galactose, and glucosamine (Harder et al., 2004). In another study, *Vibrio* sp., unidentified *Ruegeria* and *a*-*Proteobacterium* isolated from the soft coral *Dendronephthya* sp. inhibited larval settlement of *H. elegans* and *B. neritina* (Dobretsov and Qian, 2004). Natural biofilms isolated from their host, the brown alga *F. vesiculosus*, with their original composition intact, inhibited the settlement by barnacles–a protective activity which tends to be jeopardized at stressfully high and low temperature by structural shifts in the biofilm (Nasrolahi et al., 2012).

Only few antifouling compounds originating from epibiotic bacteria have been isolated and identified (Dobretsov et al., 2006b; Table 3). One of the first antifouling compounds identified as ubiquinone was isolated from *Alteromonas* sp., a marine bacterium associated with the sponge *Halichondria okadai* (Kon-ya et al., 1995). *Acinetobacter* sp., isolated from the surface of the ascidian *Stomozoa murrayi*, produces 6-bromindole-3-carbaldehyde that inhibits settlement of cyprid’s larvae in the barnacle *B. amphitrite* at concentrations of 10 mg ml^−1^ (Olguin-Uribe et al., 1997). Phenazine-1-carboxylic acid, 2-*n*-hyptyl quinol-4-one, 1-hydroxyphenazine-1-carboxylic acid, and pyolipic acid produced by the epibiotic bacterium *Pseudomonas* sp. associated with the nudibranch *Archidoris pseudoargus* inhibited *B. amphitrite* settlement (Burgess et al., 2003). Six poly-ethers A–E were isolated from *Winogradskyella poriferorum* isolated from the Bahamian sponge *Lissodendoryx isodictyalis* (Dash et al., 2009, 2011). These compounds inhibited settlement of the barnacle *B. amphitrite* and the bryozoan *B. neritina* but did not produce any adverse effects on the phenotypes of zebra fish embryos, which makes them promising candidates for antifouling applications.

Are epibiotic bacteria and their compounds able to protect their host in the natural environment? Given that “bioactivity” is concentration dependent, as we mentioned before, it is extremely difficult to simulate *in vitro* the mostly unknown *in situ* concentrations of bacterial metabolites in or on the biofilms and a clear answer is therefore not possible in studies where bacterial extracts were tested. When biofilms are tested *in vivo*, preferably in multispecies composition similar to the natural epibiotic biofilm (as e.g., in Nasrolahi et al., 2012), the answer is more straightforward. The bacteria *P. tunicata* and *Phaeobacter* sp. strain 2.10 (formerly *Roseobacter gallaeciensis*) associated with the alga *U. australis* can inhibit larval settlement at densities of 10^3^ to 10^5^ cells cm^−2^, which is similar to the densities of these bacteria under the natural conditions (Rao et al., 2007). Thus, at least in the cases of the marine macroalgae *F. vesiculosus* and *U. australis* the epibiotic biofilms seem to contribute to the host’s defense against macrofouling. It is likely that these cases are not exceptional.

#### Quorum sensing and its modulation

Quorum sensing is a cell-cell communication mechanism that allows bacteria to coordinate settlement, swarming, reproduction, biofilm formation, stress resistance, dispersal, and production of secondary metabolites (Waters and Bassler, 2005; Irie and Parsek, 2008; Steinberg et al., 2011). During this process, bacteria produce, release, and perceive small chemical signals named autoinducers. When the concentration of these signals in the environment reaches the threshold level, this triggers expression of target genes and change in the behavior of bacteria. There are various QS signaling systems used by Gram negative and Gram-positive bacteria, but the best known and characterized one is based on the production and perception of *N*-acyl homoserine lactones (AHLs) in Gram negative bacteria. Some Gram negative bacteria, like *Pseudomonas* spp. and *Vibrio* spp. produce multiple QS-signals (reviewed by Paul and Ritson-Williams, 2008; Dobretsov et al., 2009).

Most studies of settlement induction by biofilms have been realized on non-living surfaces. Their results cannot always be extended to host-epibiont interactions, as epilithic microbial communities usually differ in composition (and metabolomic activity) from epibiotic assemblages. However, if an identified settlement cue is released by epiphytic or epizooic microorganisms, an effect on settlement may at least be expected. This is the case with AHLs. Various bioactive AHL’s are generated by approximately 30% of the bacteria associated with corals (Golberg et al., 2011), as well as by microorganisms associated with sponges (Taylor et al., 2004) and seaweeds (Berger et al., 2011). Some bacteria, like *Bacillus* spp., can produce enzymes such as AHL-acylase and AHL-lactonase that hydrolyze AHL signals and make QS impossible (reviewed by Dobretsov et al., 2009). These enzymes can be used by epibiotic bacteria in order to outcompete other bacterial species.

The behavior of various seaweed-associated bacteria is affected by AHL and by inhibitors of AHL-mediated QS (Maximilien et al., 1998), which also confirms that marine epibiotic communities produce and use AHL signals. AHL signals generated by artificial (Joint et al., 2002) and natural (Tait et al., 2009) biofilms attract zoospores of the green macroalga *Ulva intestinalis*, a facultative epiphyte on various seaweeds and eelgrass. There is also evidence that bacterially produced AHL’s modulate the interaction of the red alga *Gracilaria chilensis* and its red algal epiphyte *Acrochaetium* sp. by controlling spore release in the latter (Weinberger et al., 2007). Similarly, out of 96 bacterial strains isolated from the brown alga *Colpomenia sinuosa* 12% inhibited AHL-mediated QS (Kanagasabhapathy et al., 2009) that induces spore release and spore settlement of certain algal epiphytes. The role of QS in controlling infections is just emerging (Campbell et al., 2011).

Given the important role of QS in bacterial and bacteria-alga signaling it is not surprising that some basibionts have learned to suppress this communication in order to control epibiosis and infections (reviewed by Dobretsov et al., 2009; Goecke et al., 2010; Steinberg et al., 2011).

### Feeding modulation

The modulation (enhancement, reduction) of feeding by macroepibionts is well investigated (e.g., Wahl, 2008a). In contrast, to which extent epibiotic biofilms or other associated bacteria contribute to the regulation of feeding on their host is largely unknown. Many marine organisms possess secondary metabolites of various functions that are structurally similar to known microbial metabolites, but so far only relatively few studies have rigorously demonstrated microbial production of these metabolites (Piel, 2004, 2009). Although chemical defense against consumers is a common trait among seaweeds, sponges, bryozoans, tunicates, and other members of sessile, soft-bodied marine taxa (Paul and Ritson-Williams, 2008; Paul et al., 2011), the evidence for contribution from sym- or epibiotic bacteria to this defense is limited. An example of a defensive symbiosis was discovered on coral reefs in Papua New Guinea, where epibiotic microbial communities dominated by Cyanobacteria of the genus *Synechococcus* protect their host–isopods of the genus *Santia*–from fish predators (Lindquist et al., 2005). The isopods consume these photosymbionts that live on their surface and warrant their growth by staying in sunlit areas which should make them more vulnerable to fish predators, especially so because the epibionts are brightly pigmented. However, the epibionts produce chemical deterrents that strongly detract the predators.

Not only mutualistic, but also antagonistic microorganisms in some cases modulate the interactions between marine organisms and their consumers. For example, the activation of innate immune responses in *F. vesiculosus* through challenge with cell wall matrix degradation products resulted in a reduction of palatability to the omnivorous isopod *Idotea baltica* (Kruse et al., 2009). Cell wall matrix degradation without simultaneous tissue destruction obviously results from pathogen attacks rather than predator attacks, and the innate immune system of brown seaweeds is known to fend off opportunistic cell wall macerating pathogens (Küpper et al., 2002). In brown seaweeds pathogen attacks may thus induce not only anti-pathogen, but also anti-herbivore defenses. The scarcity of reports on effects of epibiotic bacteria on the consumers of hosts should, however, not be interpreted as a scarcity of such effects.

### Biofilm-driven modulation of infections

Various diseases of marine organisms are caused by opportunistic pathogens. For example, Vibrionaceae are well known as opportunistic pathogens in algae (Weinberger et al., 1994; Largo et al., 1995), crustaceans (Selvin and Lipton, 2003), mollusks (Liu et al., 2001; Paul-Pont et al., 2010), and fish (Martinez Diaz and Anguas Velez, 2002; Tian et al., 2008; Ye et al., 2008; Zhao et al., 2010). Under most circumstances, these Vibrionaceae are harmless to their hosts, but under specific–stressful–conditions they may turn virulent. Antibiotically active marine epibionts have successfully been tested as control agents of opportunistic pathogens in fish aquaculture (Planas et al., 2006). Correlative studies have repeatedly suggested that a structural shift in associated bacteria co-occurs with an infection of the host (e.g., Frias-Lopez et al., 2002; Pantos et al., 2003). Only very recently a more mechanistic approach to the host–epibacteria–pathogen interactions in a red macroalga was undertaken showing that warming stress led to a reduction in QS-suppressing furanones resulting in enhanced infections and bleaching of the host (Steinberg et al., 2011). Nonetheless, outside the few mentioned studies, relatively little hard evidence of symbiotic microbes defending their host against microbial infection in non-artificial systems has arisen since early ground breaking studies (Gil-Turnes et al., 1989; Gil-Turnes and Fenical, 1992), who demonstrated that bacterial epibionts protect crustacean eggs from infection by an oomycetic pathogen. This may perhaps simply be due to a pertaining lack of studies. However, a clear cut distinction of environmental impact upon the three cohorts of mutualistic, antagonistic, and commensalistic microorganisms that are associated with a host is extremely difficult under most conditions, which may also explain the rarity of published data.

## Environmental Factors Affecting Microbial Epibiosis

Biofilms functionally represent a new “skin” to the host organism and we have shown in the foregoing discussion that this skin has some potential to modulate the host’s abiotic and biotic interactions. The composition of epibiotic biofilms may be host-specific to some degree (e.g., Lachnit et al., 2010; Goecke, 2011). Any compositional change in the biofilm may, but not necessarily does (Burke et al., 2011b), affect biofilm functions and, ultimately, the ecology of the host. Environmental changes (seasonality, disturbances, stress gradients, climate change) may affect biofilm composition directly or via physiological responses of the host which, in turn, lead to changes of the conditions in the boundary layer microhabitat.

Stress in its widest meaning, i.e., any combination of environmental variables reducing a species’ performance (Wahl et al., 2011), is omnipresent, at least with regard to single variables. Rarely are all requirements of an organism–temperature, light, salinity, pH, nutrients, etc.–at their optimum value. Species are adapted to tolerate sporadic or rhythmic deviation from optimum settings which are typical for their habitat (e.g., Sanford and Kelly, 2010). However, this tolerance often goes along with a decrease in performance. If performance shifts are unequal among interaction species, these interactions will shift constituting an ecological lever which buffers or enhances the impact of environmental stress (Wahl, 2008b; Fabricius et al., 2011; Monaco and Helmuth, 2011). Environmental stress will affect the interaction between host and biofilm, but also among the components of the latter (Harder et al., 2012).

For non-epibiotic biofilms it has been shown repeatedly that they may vary among habitats and season even at small spatial scales (e.g., Thompson et al., 2005; Hung et al., 2007; Anderson-Glenna et al., 2008). The abundance and composition of a biofilm on a given substratum is the combined result of the regional composition of the pool of potential colonizers, selective recruitment onto a surface, the activity of consumers, substrate characteristics, and abiotic factors (temperature, salinity, nutrients, irradiation, pH, etc.) which determine the presence and performance of single strains and the interactions among the strains (e.g., Railkin, 2004). Composition and density of the pool vary seasonally and with an exchange of water body (e.g., upwelling). Selective recruitment onto a living surface will depend on the surface’s properties (e.g., nutrients, defenses). Succession of the epibiotic biofilm community is driven by continued recruitment and interaction among biofilm components which in turn are determined by conditions in the boundary layer and activities of the host.

While the environmental control on bacterioplankton and on non-epibiotic biofilms is well studied, we know substantially less on how environmentally driven changes in the host’s performance affect epibiotic biofilm composition and their role in the host’s ecology. There is some evidence that under warming stress the prevalence of diseases increases (e.g., Ainsworth and Hoegh-Guldberg, 2009; Mydlarz and McGinty, 2010; Campbell et al., 2011). Either pathogens become more abundant, or biofilm components turn virulent, or the defensive capacities of biofilm and/or host are weakened. Grimes (2002) and Vezzulli et al. (2012) presented data that hint at increasing abundance and virulence of bacterial opportunists belonging to the Vibrionaceae in coastal waters during the last decades and suggested a link with global warming. Numerous bacterial strains that are associated with marine organisms and form biofilms on their surfaces are known to produce antibiotics (see section “Modulation of bacterial settlement by epibiotic bacteria”) and there is increasing evidence that these microorganisms contribute significantly to the host’s resistance against macro- and micro-foulers (see section “Modulation of eukaryote settlement by epibiotic bacteria”). In this light a relevant contribution of the same bacteria to the host’s resistance against opportunistic or even obligate microbial pathogens appears as possible.

Interactive effects of opportunistic pathogens, mutualistic microorganisms, and abiotic stress have been observed in corals (Ben-Haim, 2003; Bourne et al., 2007; Sussman et al., 2008; Sunagawa et al., 2009), in which bleaching-related disease symptoms appear after shifts in the bacterial community composition toward a strongly increased abundance of Vibrionaceae and Alteromonadaceae (Bourne et al., 2007). Under non-stress conditions these potential pathogens are suppressed by antibiotics secreted by beneficial bacteria in the coral mucus (Ritchie, 2006). Under temperature stress the density of beneficial bacteria decreases, which is correlated with a loss of the protective properties of the mucus. Apparently warming triggers a primary loss of protective bacteria and/or a stimulation of overgrowth by non-protective and pathogenic commensals that are characterized by a strong proteolytic activity (Sussman et al., 2008). It is probably this shift toward pathogenic bacteria that prevents the recovery of corals from temperature stress.

Seasonal warming increases the density of bacteria, pathogenic or not, by a factor 10 in the Baltic Sea (0.45 × 10^6^ per ml^−1^ in winter and 5.67 × 10^6^ per ml in summer, Zimmermann, 1977). At the same time, the phylogenetic composition of this pool varies substantially among seasons (Lachnit et al., 2010; Koskinen et al., 2010). During a warming event (and presumably in the course of climate change) the density, phenology, activity, and composition of bacterioplankton (i.e., the pool of potential colonizers) change (e.g., Hoppe et al., 2008). As the level of activity as well as physiological status of potential host organisms is affected by environmental conditions, we can expect their surface properties (fouling modulating metabolites, exudates, ${\text{O}}_{2}^{-}$, and pH values) to vary with abiotic conditions. It is not surprising then that–driven by these two factors or more–the epibiotic bacterial community on a given host species may vary strongly with season (e.g., Lachnit et al., 2010). To some extent, the composition of biofilms among co-occurring conspecific hosts also differs (Lachnit et al., 2010; Burke et al., 2011b) which may or may not lead to altered functioning of the biofilms. Functional properties of and functional differences among epibiotic biofilms are severely understudied. We do not really know what an epibiotic microorganism “does,” e.g., which compounds it anabolizes, or which of the compounds transiting between host and environment it catabolizes or transforms to new compounds. Environmental stress, i.e., a particularly strong deviation of one or more variables from an organism’s optimum, may affect the biofilm bacteria directly (see above) or indirectly via shifts in the host’s defenses or exudate quantity or quality. As a functional analogy, indirect stress effects on the intestinal biofilm of mammals with a multitude of possible health issues are well studied (e.g., Nettelbladt et al., 2003). Adverse environmental conditions such has heat waves or low light may affect the efficacy of chemical antimicrofouling defenses in the bladder wrack *F. vesiculosus* (Rohde et al., 2008; Wahl et al., 2010). Under experimental stress (warming, shading, desalination) the composition of the epibiotic biofilm on the bladder wrack re-organizes (S. Stratil, GEOMAR, pers. comm.). This may have direct consequences for *Fucus* if the bacteria involved in this change provide vitamins or nutrients, or deliver other goods to the host, an aspect unstudied for this host species, but established for the related *F. spiralis* (Fries, 1977, 1982, 1993). However, the indirect effects may be as large or larger. We have mentioned earlier (see “Fouling modulation by epibiotic biofilms”) that some bacteria epibiotic on *Fucus* hinder further fouling by other bacteria or by barnacles (Nasrolahi et al., 2012). There are reports on epibiotic bacteria suppressing infections (Reid et al., 2001) or causing them (Wang et al., 2008; Fernandes et al., 2011). Although to be expected, a modulation by biofilms of consumption pressure on the host has not yet been investigated. These examples, and those given earlier in this article, show that a compositional shift in the epibiotic biofilm probably is ecologically not trivial. Depending whether beneficial or detrimental strains change in abundance in the biofilms, such a re-structuring under stress may buffer or enhance the more direct stress effects on the host. An even more direct response to environmental shifts than re-structuring of the biofilm could be metabolic shifts within a structurally stable biofilm with its components doing more, less, or different things than under another set of environmental variables (e.g., Steinberg et al., 2011).

Apart from the local stress regimes that native organisms are expected to be adapted to, a species may be subject to stress gradients in space and time. Stress gradients in space can be found along the distributional axis from core to margin of a host species’ range, or when a species is translocated during a bioinvasion process. Stress gradients in time are associated with global change. As the coastal oceans are gradually shifting toward a warmer, nutrient richer, less oxygenated, sourer status, conditions in a given locality may turn more stressful. In addition, the introduction of alien species may represent novel interactions for the host. Bioinvasion research in the past has focused on macroorganisms. However, it was shown that invasive algae may carry along their associated biofilm (Meusnier et al., 2001) and that huge amounts of allochthonous microorganisms are continuously imported via ballast water and biofilms on moving artificial substrata such as ships (Drake et al., 2007) and, presumably but unexplored, drifting litter. Epibiotic bacteria may hitchhike over vast distances with their host (e.g., Grossart et al., 2010). Since the conditions in the boundary layer on the host’s surface, the microhabitat of the epibiotic bacteria, is very much controlled by the host, attached bacteria can be seen as traveling in a space craft across potentially adverse outside conditions.

With increasing stress on the host and/or the epibiotic bacteria both the identity of the biofilm components may change, as well as their behavior, turning neutral or beneficial epibionts into pathogens for instance (e.g., Pruzzo et al., 2008; Feeding Modulation, but, see Burke et al., 2011a). Structural and/or functional changes in the biofilm may buffer or enhance environmental stress on the host.

## Conclusion and Suggestions

What do we know?

1. Biofilm bacteria and free-living bacteria are two states of aggregation of one regional pool of bacteria. Through attachment-detachment cycles there is intense exchange between the two compartments. In the biofilm state, interactions, metabolism, reproduction, and genetic exchange are substantially accelerated compared to the free-living state. Biofilms form at interphases, mostly solid/liquid but also liquid/gas or liquid/liquid (of different densities).
2. All marine organisms bear epibiotic biofilms which range from sparse to dense and from monospecific to highly diverse.
3. These epibiotic biofilms have a huge potential to affect the biology, ecology, and fitness of their host. Many direct and indirect effects of epibiotic biofilms have been described, many more can be expected to exist (Figure 2).
4. Density and composition of epibiotic biofilms vary at different scales: among host species, among conspecific host individuals, among body regions of a host individual, among habitats, and among seasons. Structural differences among biofilms may or may not affect their function.

What we need to explore:

1. Metabolomics: newly emerging techniques such as DESI-MS coupled to MS-MS allow characterizing the surface chemical landscape of a biofilm, i.e., the compounds produced by the net metabolism of the epibacterial community (e.g., Prince and Pohnert, 2010; Nylund et al., 2011; Goulitquer et al., 2012). This chemical landscape should be characterized for the same biofilms under varying environmental conditions and host activities to asses the scope of metabolome fluctuations. Structure analysis of single compounds may allow searching for described functions.
2. “Soft” surface extraction techniques (Nylund et al., 2007; Lachnit et al., 2010; Saha et al., 2011) and non-intrusive analytical techniques (e.g., confocal resonance Rahman spectroscopy; Grosser et al., 2012) allow for the isolation and analysis of compounds in the boundary layer. Bioassays should be used to verify activities of whole or fractionated extracts against foulers, consumers, pathogens.
3. The combination of the first two approaches will shed light on the relationship between structure and function of epibacterial communities. This approach is more direct and more powerful than the metagenomics of functional genes (Burke et al., 2011b). It may help resolve the central question to which extent the observed structural differences in biofilms at numerous spatial and temporal scales are associated with functional differences. After all it is the function of the biofilm which matters for the host and the organisms interacting with it, not the identity of the biofilm components.
4. New techniques should be optimized to separate host and biofilm. This would permit to assess, at least for a short while, how the two components fare in the absence of the partner component. Any change in performance following the separation could deliver valuable hints at their interactions when united.
5. Once the ecological value of epibacterial communities is defined we may want to know to which degree and how a host can influence community services of its bacterial biofilm–via its composition and its activity. It is conceivable that a host chemically promotes fouling by beneficial strains, or by strains which in turn promote the establishment of a beneficial biofilm. Furthermore, the host could influence biofilm activities by exuding certain nutrients or infochemical (including QS active compounds).
6. Particularly understudied is the role of epibiotic biofilms for infection and disease of the host. When and how do biofilms repel pathogens and parasites, when do they stop doing this, when, and why do biofilm compounds switch from beneficial or neutral to pathogenic?
7. If certain strains are particularly beneficial for a host it would be of selective advantage if they were transmitted vertically on propagules or vegetative fragments, or horizontally on the surface of mesograzers. Attachment-detachment cycles permitting such hitchhiking and “contamination” have been described for other systems (Grossart et al., 2010).
8. Notwithstanding the general bias of this review in favor of the host-centered perspective it should be considered here that such vertical and horizontal transmission among conspecific hosts could benefit epibiotic strains by giving them a head start on a new substratum.
9. Widely neglected so far is the epibacterial perspective. Grossart and Tang (2010) have described how aggregation in biofilms affects bacterial ecology and evolution. But the specific significance of associating with a sessile host is understudied. In which regard do epibacteria benefit from host exudates, from the host-controlled conditions in the boundary layer and from the fact that they lead a stationary instead of a drifting way of life.
10. Finally, we recommend to progress from a compartmentalized view (host and epibacteria interacting with each other) to a more holistic view which recognizes that the holobiont (host with associated bacteria) is essentially inseparable physically and functionally and, perhaps, even evolutionarily.

## Conflict of Interest Statement

The authors declare that the research was conducted in the absence of any commercial or financial relationships that could be construed as a potential conflict of interest.
